# Association of common IL-10 promoter gene variants with the susceptibility to head and neck cancer in Tunisia

**DOI:** 10.3906/sag-1805-21

**Published:** 2019-02-11

**Authors:** Lamia MAKNI, Cherif BEN HAMDA, Abrar K. AL-ANSARI, Oussema SOUIAI, Ezzedine GAZOUANI, Amel MEZLINI, Wassim Y. ALMAWI, Besma YACOUBI-LOUESLATI

**Affiliations:** 1 Laboratory of Mycology, Pathologies and Biomarkers (LR16ES05), Faculty of Sciences of Tunis, El Manar University, Tunis Tunisia; 2 Laboratory of Bioinformatics, Biomathematics and Biostatistics, Institute Pasteur of Tunis, Tunis Tunisia; 3 Laboratory of Immunology, Military Hospital of Tunis, Tunis Tunisia; 4 Salah Azeiz Oncology Institute, Tunis Tunisia

**Keywords:** Head and neck cancer, interleukin-10, laryngeal cancer, nasopharyngeal cancer, Tunisia

## Abstract

**Background/aim:**

We investigated the association of three IL-10 promoter single-nucleotide polymorphisms and altered IL-10 plasma levels with the risk of head and neck cancer (HNC).

**Materials and methods:**

Study subjects comprised 194 HNC patients [137 nasopharyngeal cancer (NPC) and 57 laryngeal cancer (LC)], and 263 healthy controls. Genotyping of rs1800896 (-1082A>G), rs1800871 (-819C>T), and rs1800872 (-592A>C) IL-10 variants was performed by real-time PCR; IL-10 levels were measured by enzyme amplified immuno sensitivity assay (EAISA).

**Results:**

Carriage of rs1800896 A/A genotype was more frequent in the HNC and NPC cases, but was less frequent in the controls than the LC patients. Significant differences in IL-10 levels were observed between the rs1800896A/G genotype-carrying NPC patients and the controls. Positive association with NPC and LC was observed for rs1800871C/C, and carriage of rs1800872A/A genotype, and A allele were associated with higher risk of HNC and NPC, but not LC. GT rs1800896-rs1800871 haplotype was more frequent among the HNC and NPC patients than the controls in contrast to GC haplotype, which has a protective effect. Positive association was found between TA haplotype and LC.

**Conclusion:**

Our results demonstrate that IL-10-1082, IL-10-819, and IL-10-592 variants, and haplotypes GC and GT constitute biomarkers for early detection of HNC, especially NPC subtype. IL-10 -819T/C and TA haplotype may be used as biomarkers for early detection of LC.

## 1. Introduction

Recent evidence supports increased head and neck cancer (HNC) incidence worldwide, with estimated 400,000–600,000 new cases per year, and 223,000–300,000 annual deaths (1,2). HNC arises from malignant transformation of upper respiratory tract epithelial cells (3), and nasopharyngeal carcinoma (NPC) and laryngeal cancer (LC) are the most prevalent forms of HNC (4). Although NPC and LC are anatomically and histologically related, they differ in the pathogenesis, biology, treatment, mortality, and morbidity, as well as geographic and ethnic incidence (5–7). HNCs are multifactorial malignancies, which are influenced by environmental, viral, immunogenetic, and lifestyle risk factors (8–10). However, few studies addressed the implication of these factors in the variability of and the relative susceptibility to NPC and LC within different populations. Several reports documented the contribution of altered balance of proinflammatory and antiinflammatory mechanisms in HNC development, but with mixed outcome (11,12). As an immunosuppressive cytokine, interleukin-10 (IL-10) was described as a key player in the pathogenesis and progression of NPC and related malignancies (13).

Located on chromosome 1 (1q31–q32), human *IL-10 *gene consists of five exons and four introns (14), and several single nucleotide polymorphisms (SNP) were identified in *IL-10* promoter region (15). Of these, rs1800896 (-1082A/G), rs1800871 (-819C/T), and rs1800872 (-592A/C) were the most investigated (16,17). Previous studies documented the association of these *IL-10 *variants with cancers (18–20), including NPC (21), but with inconclusive findings, and an ethnic contribution to this association appeared likely. As these *IL-10* promoter variants rs1800896 (-1082 A/G), rs1800871(-819 C/T), and rs1800872 (-592 A/C) modulate IL-10 serum levels, and hence HNC susceptibility, the purpose of the present study was to evaluate these three *IL-10* promoter polymorphisms and plasma IL-10 levels as potential biomarkers for HNC.

## 2. Materials and methods

### 2.1. Study subjects 

Between November 2012 and October 2015, 194 HNC cases were consecutively recruited from Salah Azaiz Oncology Institute (SAI, Tunisia). All HNC cases originated from North Tunisia, of whom 137 presented with histologically confirmed nondifferentiated carcinoma of nasopharyngeal type (UCNT), and the remaining 57 presenting with histologically confirmed squamous cell carcinoma (SCC) of laryngeal type. Cancer diagnosis was established by clinical and biopsy examination, and confirmed by an SAI senior pathologist. Clinical data were obtained by unified questionnaire, personal interviews, and review of case records. All subjects were asked to sign a consent form, agreeing to participate in the study, after all institutional ethical requirements were met. The control group consisted of 263 unrelated blood donors, who reported no personal or family history of cancer, or other chronic illness. 

### 2.2. DNA extraction 

Peripheral blood samples (5 mL) of patients and control subjects were collected in EDTA-containing tubes. Genomic DNA was extracted from buffy coat layer using QIAamp® DNA blood Mini Kit, according to the instruction of the manufacturer (Qiagen GmbH, Hilden, Germany). DNA was stored in nuclease-free water at 4 °C pending analysis.

### 2.3. IL-10 plasma levels 

Blood samples were collected from patients and controls in plain tubes. Plasma was separated by centrifugation at 2000 ×*g* for 15 min at 4 °C, and stored as 250 µL aliquots −80 °C pending analysis. IL-10 levels were measured using a commercial EAISA kit, according to the manufacturer’s instructions (DIA source Immuno Assay, Louvain-la-Neuve, Belgium). The ELISA reader-controlling software (SoftMax) was used in processing raw absorbance values into a standard curve, from which IL-10 concentrations (pg/mL) were obtained.

### 2.4. IL-10 genotyping 

The three *IL-10* gene polymorphisms studied, i.e. rs1800872, rs1800871, and rs1800896, were selected due to their minor allele frequency (MAF) of >5% in Caucasians, and previous association with HNC development. *IL-10 *genotyping was performed using the allelic (VIC- and FAM-labeled) discrimination method. Assay-on-demand TaqMan genotyping assays were ordered from Applied Biosystems (Foster City, CA, USA): C_1747363_10 (rs1800872), C_1747362_10 (rs1800871), and C_1747360_10 (rs1800896). The reaction was performed in 6 µL volume on StepOne Plus real-time PCR systems, according to the manufacturer’s instructions (Applied Biosystems). Replicate blinded quality control samples were included to assess genotyping reproducibility; the concordance was >99%. 

### 2.5. Statistical analysis 

Statistical analysis was performed with R software for windows and R studio program (www.rstudio.com), version 3.2.3. The characteristics of the study subjects were reported as mean for age, medians for IL-10 level, and relative frequencies for sex, tobacco and alcohol consumption, and histological type. The effect of *IL-10* polymorphisms on IL-10 plasma levels, as well as HNC, NPC, and LC were evaluated by setting homozygous major allele genotype as reference; subsequent analyses were performed using one-way ANOVA. Chi-quare test was used in comparing categorical data and Fisher exact test for low number. Receiver operating characteristic (ROC) curve was used to determine sensitivity and specificity; P < 0.05 was considered statistically significant. Linkage disequilibrium and haplotype reconstruction were performed using Haploview 4.2 (www.broadinstitute.org) software and SNPstats (u299e), which also tested for departure from Hardy–Weinberg equilibrium (HWE).

### 2.6. Ethics

The study protocol was approved by the Ethics Committee of Salah Azeiz Oncology Institute in Tunis, Tunisia.

## 3. Results

### 3.1. Study participants

Table 1 summarizes the demographic and clinical characteristics of HNC patients and control subjects. Study subjects comprised 194 patients (137 with NPC, mean age 48 years, and 57 with LC, mean age 46.31 years), and 263 control subjects (mean age 49.96 years). The median IL-10 plasma level was slightly lower in the HNC patients [2.25 (0.003–39.45) pg/mL than the control subjects [4.65 (1.79–10.73) pg/mL], but was not statistically significant (P = 0.34). 

**Table 1:** Clinical characteristics of HNC, NPC, and LC patients and the healthy controls.

Characteristics	Controlsn = 263	HNCn = 194	OR1	(95% CI)	NPC n=137	OR2	(95% CI)	LCn = 57	OR3	(95% CI)	P-value
Female/male	171/92	45/149	6.154	4.077–9.431	39/98	4.671	3.001–7.383	6/51	15.799	7.032-42.367	<0.001
Age (years)	49.96	49.23	0.995	0.981–1.010	48	0.977	0.960–0.993	46.31	1.049	1.022–1.077	0.54
Tobacco	56/207	126/68	6.849	4.539–10.467	75/62	4.471	2.870–7.032	51/6	31.420	13.786–85.169	<0.001
Alcohol	39/224	76/118	3.669	2.382–5.821	44/93	2.717	1.660–4.470	32/25	7.352	3.963–13.858	<0.001
Histology											
UCNT	NA	137	-	-	137	-	-	-	-	-	-
SCC	NA	57	-	-	-	-	-	57	-	-	-
IL-10 level (pg/mL)	4.650	2.250	0.969	0.908–1.047	2.250	0.944	0.863–1.026	3.120	1.008	0.917–1.129	0.34

HNC: head and neck cancer; n: number of subjects; NPC: nasopharyngeal carcinoma; LC: laryngeal cancer; OR1: odds rate between the HNC patients and the controls, OR2 : odds rate between the NPC patients and the controls; OR3: odds rate between the LC patients and the controls; CI: confidence interval; P-value: one way ANOVA; Age: per year; Tobacco: smokers/nonsmokers; Alcohol: drinkers/nondrinkers; UCNT: undifferentiated carcinoma nasopharynx type; SCC: squamous cell carcinoma; NA: not applicable; IL-10 level : (pg/mL).

### 3.2. Association between IL-10 polymorphisms and HNC

The distribution of *IL-10* -1082A/G, -819C/T, and -592C/A genotypes did not deviate from HWE (P* > *0.05). Table 2 lists the frequencies of the genotype profiles of the tested SNPs, first between the HNC patients and controls, second between the NPC patients and the controls, and third between the cases with LC and the controls. Univariate and multivariate analyses confirmed significant differences between the HNC or NPC patients and the controls in the distribution of the three tested *IL-10 *SNPs. Significant differences were observed between the LC patients and the controls in the distribution of rs1800896 (-1082A>G) and rs1800871 (-819C>T). 

**Table 2:** Association between IL-10 genotypes and the development of head and neck, nasopharyngeal and laryngeal cancers.

SNPs	HWE	P1	OR	P1*	P2	OR	P2*	P3	OR	P3*
rs1800896	0.253									
A/A		reference	1.00	0.000	reference	1.00	0.001	reference	1.00	
A/G		0.028	1.58 [1.05–2.40]		0.007	1.88 [1.18–3.03]		0.767	1.09 [0.59–2.02]	0.036
G/G		0.064	0.57 [0.31–1.02]		0.391	0.74 [0.38–1.42]		0.020	0.28 [0.08–0.78]	
G		0.777	0.94[0.69–1.28]		0.978	1.01[ 0.72–1.42]		0.3638	0.77[0.48– 1.25]	
rs1800871	0.707									
C/C		reference	1.00	0.019	reference	1.00	0.042	reference	1.00	
C/T		0.232	1.26 [0.85–1.87]		0.936	0.98 [0.63–1.52]		0.006	2.35 [1.27–4.48]	0.009
T/T		0.005	2.67 [1.32–5.59]		0.016	2.50 [1.16–5.44]		0.021	3.33 [1.07–9.50]	
T		0.159	1.27[0.92–1.76]		0.472	1.16[0.80–1.67]		0.087	1.54[0.96– 2.47]	
rs1800872	0.830									
C/C		reference	1.00	0.026	reference	1.00	0.016	reference	1.00	
C/A		0.998	1.00 [0.67–1.47]		0.384	0.82 [0.52–1.27]		0.139	1.58 [0.86–2.97]	0.118
A/A		0.009	2.51 [1.24–5.24]		0.017	2.45 [1.15–5.32]		0.060	2.70 [0.87–7.61]	
A		0.023	1.15 [0.83–1.58]		0.013	1.08 [0.75–1.55]		0.291	1.31 [0.82–2.11]	

SNP: single nucleotide polymorphisms; OR: odds ratio; CI: confidence interval; Significant associations P < 0.05 are indicated in bold; P1 value: chi-square test between the HNC patients and the controls; P1*: one-way ANOVA between the HNC and the controls; P2 value: chi-square test between the NPC patients and the controls; P2*: one-way ANOVA between the NPC patients and the controls; P3 value: chi-square test between the LC patients and the controls; P3*: one-way ANOVA between the LC patients and the controls.

The distribution of rs1800896 A/A genotype was significantly different between HNC cases and control subject [P < 0.001, OR (95% CI) = 1.58 (1.05–2.40)], and between the NPC patients and controls [P = 0.001, OR (95% CI) = 1.88 (1.18–3.03)]. Negative association was observed for this genotype between th LC cases and the controls [P = 0.036, OR (95% CI) = 0.28 (0.08–0.78)]. A positive association was noted in the association of rs1800871 C/C genotype with NPC [P = 0.042, OR (95% CI) = 2.50 (1.16–5.44)] and LC [P = 0.009, OR (95% CI) = 3.33 (1.07–9.50)] susceptibility. In addition, rs1800872 A/A genotype was associated with higher risk of HNC [P = 0.026, OR (95% CI) = 2.51 (1.24–5.24)] and NPC P = 0.016, OR (95% CI) = 2.45 (1.15–5.32)], but not LC. In addition, rs1800872 minor (A) allele frequency was associated with higher risk of HNC and NPC.

### 3.3. Plasma IL-10 level analysis 

**Figure 1:**
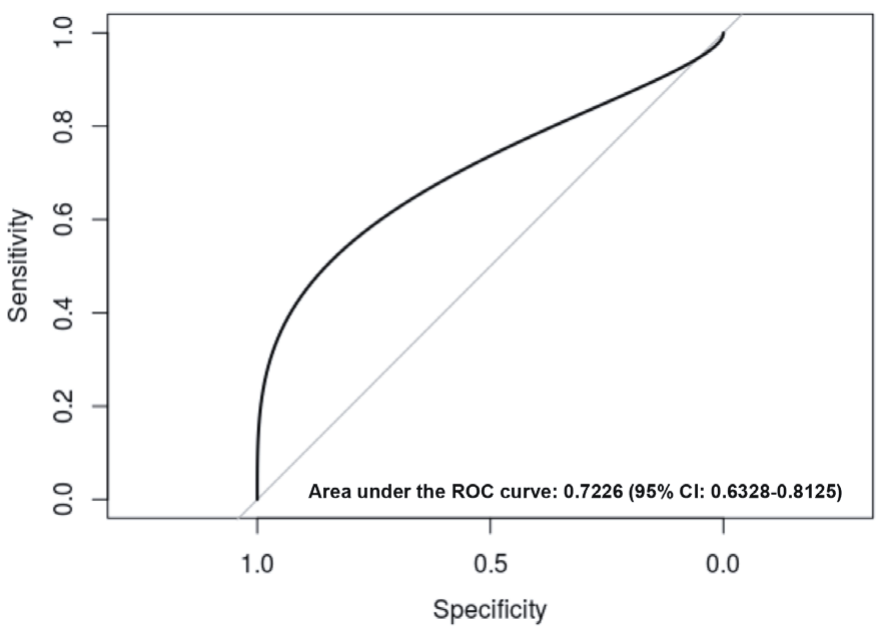
Sensitivity and specificity of IL-10 level measurment. Receiver operating characteristic (ROC) curve on binary logistic regression and discriminant classification analysis for the HNC and the control groups. Sensitivity and specificity were assessed by measuring the area under the curve.

**Figure 2:**
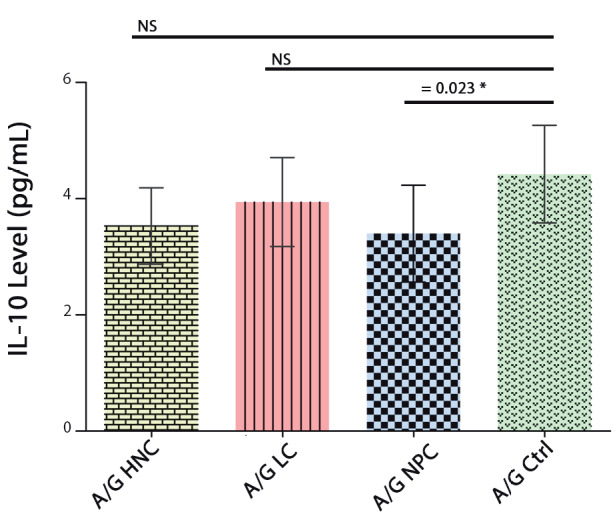
Level of IL-10 in the HNC, NPC, and LC patients and the controls AG genotype of rs1800896. HNC: head and neck cancer; NPC: nasopharyngeal cancer; LC: laryngeal cancer; Ctrl: control. NS: not significant.

Plasma IL-10 levels in the HNC patients and the control subjects were next determined, and ROC analysis was performed to assess sensitivity and specificity. The area under the curve was 0.72 (Figure 1), suggesting insufficient sensitivity and specificity of EAISA determinations. HNC cases and controls were stratified according to specific *IL-10* genotypes, and plasma IL-10 concentrations were determined for specific *IL-10 *genotypes in the patients and the controls. Results from Figure 2 and Table 3 demonstrate a significant difference in IL-10 plasma levels between -1082 A/G (rs1800896) NPC patients and control subjects genotype carriers (P = 0.023).

**Table 3:** Genotype distribution and serum IL-10 levels.

SNP/genotype	IL-10 level (pg/mL)		P-value
	Cases	Controls		
	Means ± SD	Means ± SD		
IL-10-1082 A/G			
AA	2.46 ± 2.47	4.06 ± 2.18	0.121^1^
AG	3.59 ± 6.41	4.44 ± 2.78	0.023^1^
GG	3.68 ± 4.05	6.05 ± 2.38	0.155^2^
AA vs. AG+GG			0.792^3^

^1O^btained using the Mann–Whitney U test, 2obtained using the Student’s t-test, 3comparison between IL-10 levels in the carriers and those in the noncarriers.

### 3.4. Haplotype analysis

Haploview analysis revealed high linkage disequilibrium (LD) between rs1800871 and rs1800896 (0.72), but weak LD between rs1800872 and the two other SNPs (LD = 0.46 and 0.45). *IL-10* haplotypes were constructed based on rs1800871 and rs1800896 minor allele frequencies, with four haplotypes (with a frequency >1%) were found. Results from Table 4 demonstrated enrichment of the two minor alleles GT haplotype among the HNC and NPC patients than the controls, thereby assigning disease susceptibility nature to this haplotype [P = 0.002, OR (95% CI) = 2.98 (1.48–6.02); P < 0.001, OR (95% CI) = 3.62 (1.75–7.48)], respectively. The distribution of the other haplotypes was comparable between the HNC and the controls. However, GC haplotype appears to be protective of HNC [P = 0.038, OR (95% CI) = 0.69 (0.49–0.98)] and NPC subtype [P = 0.021, OR (95% CI) = 0.64 (0.43–0.95)]. Furthermore, a positive association was found between TA haplotype and LC [P = 0.03, OR (95% CI) = 2.25 (1.32–3.83)].

**Table 4:** Two-loci IL-10 haplotype distribution in head and neck, nasopharyngeal and laryngeal cancer cases and controls.

	HNC				NPC			LC		
Haplotype^1^	Cases	Controls	P	OR (95% CI)	Cases	P^2^	OR (95%CI)	Cases	P^3^	OR (95% CI)
AC	0.379	0.352	–		0.397	_		0.311	0.67	1.12 (0.66–1.89)
GC	0.283	0.387	0.038	0.69 (0.49–0.98)	0.288	0.0219	0.64 (0.43–0.95)	0.294	–	
AT	0.229	0.225	0.81	0.95 (0.64–1.43)	0.182	0.12	0.68 (0.42–1.11)	0.364	0.03	2.25 (1.32–3.83)
G T	0.108	0.034	0.002	2.98 (1.48–6.02)	0.131	<0.001	3.62 (1.75–7.48)	0.030	0.83	(0.22–6.49)

HNC: head and neck cancer; n: number of subjects; NPC: nasopharyngeal carcinoma; LC: laryngeal cancer. 1: two-locus rs180087-rs1800896 haplotype; underlined bold indicates minor allele; 2: nasopharyngeal cancer versus controls; 3: laryngeal cancer versus controls

## 4. Discussion

Increasing evidence implicated imbalance of pro- and antiinflammatory cytokines in the persistence of local inflammation in HNC patients, including NPC and LC, of which IL-10 was hypothesized to be a key determinant in HNC growth (22,23). Recent studies suggested that genetic polymorphisms in pro- and antiinflammatory cytokines contribute to the pathogenesis of several malignancies, including HNC (24,25). This prompted the speculation that cytokine gene variants may serve as potential biomarkers for NPC detection, especially in the early stages. This incited us to investigate the possible relationship between common* IL-10 *promoter variants -1082A/G (rs1800896), -819C/T (rs1800871), and -592C/A (rs1800872), and the risk of HNC in Tunisians.

A positive association was observed between -1082A/G (rs1800896) polymorphism and HNC and NPC risk. This was in agreement with two recent metaanalyses. The first one carried out on nine studies involving 2258 HNC patients and 2887 control subjects reported an OR of 1.64 when the -1082A/G genotype carriers were compared to those of -1082A/A (24). The second metaanalysis, including four NPC case-control studies, revealed that carriage of -1082A/G and -1082A/A genotypes were associated with 1.77-fold elevated risk of NPC, when compared with the homozygous G/G genotype (21). Collectively, this supports the notion that -1082A/G *IL-10 *variant constitutes a risk factor for HNC and NPC. On the other hand, a negative association of rs1800896 A/A genotype was observed between the LC patients and the controls, suggesting a protective effect of this variant against LC development. 

*IL-10 *rs1800871 C/C genotype was associated with 2.5-fold and 3.33-fold higher risk of NPC and LC development, respectively, and carriers of rs1800872 A/A genotype were at 2.5-fold higher risk for HNC and NPC but not LC. In contrast, no statistically significant association was detected between these two *IL-10* variants and HNC and NPC according to the results of these two recent metaanalyses (21,24).

While the tested *IL-10 *variants were linked with altered IL-10 secretion in rheumatoid arthritis (17), and gastric inflammation (26), few studies investigated the association between *IL-10 *genotypes and IL-10 levels in HNC. To the best of our knowledge, this was the first study that examined the association between *IL-10 *genotypes and IL-10 levels in HNC. Although the three tested variants were previously linked with altered IL-10 secretion, our findings indicate that only rs1800896 was associated with reduced IL-10 levels. These points to the contribution of HNC-associated factors in regulating the IL-10 secretion. 

Haploview analysis revealed high LD between rs1800871 and rs1800896, but weak LD between rs1800872 and the two other *IL-10 *variants. Of the four obtained haplotypes, GT (minor) and GC were positively associated with HNC and NPC, but not with LC. In addition, AT haplotype was positively associated with LC but not with NPC. The contradictory results obtained for LC and NPC are explained by the different etiologies of these HNCs. This is supported by our recent study evaluating the effect of eight *VEGF-A* polymorphisms on LC and NPC susceptibility, which revealed that none of the tested variants significantly influenced LC risk contrary to their association with NPC, further confirming that these HNCs have different etiologies (27).

## 5. Conclusion

The present findings showed that *IL-10 *rs1800896, rs1800871, and rs1800872 variants, along with specific (2-loci) haplotypes contribute to the development of NPC. This suggests a possible role for these variants as biomarkers for early detection of HNC and especially the NPC subtype. However, *IL-10 *rs1800871 and AT haplotype may be used for detection of subjects at higher risk of LC.

## Acknowledgments

We thank all blood donors and patients with HNC who voluntarily participated in the present study. We are grateful to the staff of Salah Azaiz Oncology Institute, Tunisian Center of Maternity and Néonatalogy, and Dispenser of Ettadhamen City for their help in the collection of blood samples. 
